# A Case of Spasmodic Asthma

**Published:** 1852-01

**Authors:** H. A. Swasey

**Affiliations:** Mississippi


*A case of Spasmodic Asthma.* Reported by H. A. Swasey, M.D. of Mississippi.

[The following case, though presenting nothing very novel, accompanied a communication from the author containing so much of the right spirit, that we cheerfully give it a place as desired. The author believes that every member of the medical profession has a just claim upon his brethren for whatever knowledge they may possess in regard to their common calling, and in this spirit makes his contribution to the therapeutics of a disease, at all times interesting and often difficult to manage.—Eds.]

A case of this disease which came under my care, some time in the beginning of January last, possessed so many points of interest in its previous history, and was so promptly relieved by a somewhat novel treatment, that I have thought a record of it worth preserving.

Oscar C., æt. 10, was taken, some few years ago, with a severe chill, followed by high fever, which however soon subsided, leaving the system to all appearances free from disease. On the day following, he was attacked with "something like a fit," as his parents express it, accompanied by great dyspnœa, which continued unmitigated after the subsidence of the convulsive paroxysm.

A Physician, Dr. W., was now called in, who, finding considerable soreness and some little pain in the chest, pronounced it *pleuro-pneumonia*, and prescribed accordingly, with no appreciable benefit.

The attack, however, gradually wore off, although a shortness of breath remained, and the slightest exertion produced considerable dyspnœa. A year passed by, when, without any premonition, or assignable cause, another attack supervened, similar in almost every respect to the first. Another physician, Dr. M., was now called, who, after a *thorough* exploration, pronounced the disease "the *consequence* of a *deformed chest*," and declined, I believe, doing anything for the case. At any rate no very efficient remedies were prescribed, for the disease continued to return occasionally, though with less violence, during the next six months, when the parents of the sufferer, almost in despair, called in another physician, Dr. D., who pronounced it *asthma*, prescribed tinct. lobelia in emetic doses,—instructed the parents in the indications for its use, mode of administration, etc., and left the patient in their hands. The remedy seemed a good one, for by its use all severe attacks were warded off during a period of nearly a year and a half, when its power over the disease seemed to be entirely annulled. Ipecac. was then substituted, but it, too, was inefficient in relieving the paroxysms, which had now become so severe as to be accompanied by general convulsions.

It was in one of these convulsive attacks that I was requested to see him. When I reached Mr. C.'s, some two miles distant, the spasmodic paroxysm had subsided, but the dyspnœa was almost suffocative. The symptoms were, unmistakably, those of spasmodic asthma. I ordered an emetic of ipecac. and tinct. lobelia to be given immediately, and its prompt action insured by copious draughts of warm water. It operated finely, ejecting from the stomach a considerable quantity of mucus and crude ingesta, to the no small, but not entire relief of the patient. Chloroform was then administered by inhalation, until its soporific effects were induced. All difficulty of respiration disappeared, and the patient slept quietly.

Not daring to trust the administration of chloroform, *by inhalation*, to the hands of the parents, considering it as I do "a good servant but a bad master," I left a mixture composed of equal parts of chloroform, sul. ether, and tinct. opii cam., "to be given, 30 drops at a time, in syrup, as soon as the symptoms of the next paroxysm made their appearance. The dose to be repeated every half hour until the attack is subdued."

It has been nearly a year since this "abortive" treatment was first made use of in this case, and the parents inform me that it "acts like a charm," that a few doses of the medicine never fail to produce the desired effect.

This treatment may, of course, need further modification, according to the peculiarities of each particular case, but having used it in two or three other less severe cases of pure spasmodic asthma, with the happiest results, I am inclined to consider it a most valuable course, and one eminently worthy of more extended trial.

P. S. I would remark, in justice to the gentlemen referred to above, that they all stood high in the ranks of our profession.

